# Supplementary material for: A Fast and Minimal System to Identify Depression Using Smartphones: Explainable Machine Learning–Based Approach
Source: JMIR Form Res. 2023 Aug 10;7:e28848. doi: 10.2196/28848 (PMC10450542; doi:10.2196/28848)
Supplement: Multimedia Appendix 1 [file formative_v7i1e28848_app1.docx]

**A Minimal and Faster System to Identify Depression Through Smartphones: Explainable Machine Learning–Based Approach**

**Md Sabbir Ahmed and Nova Ahmed**

Design Inclusion and Access Lab, North South University, Bangladesh

*msg2sabbir@gmail.com*, *nova.ahmed@northsouth.edu*

# **Data Collection Tool**


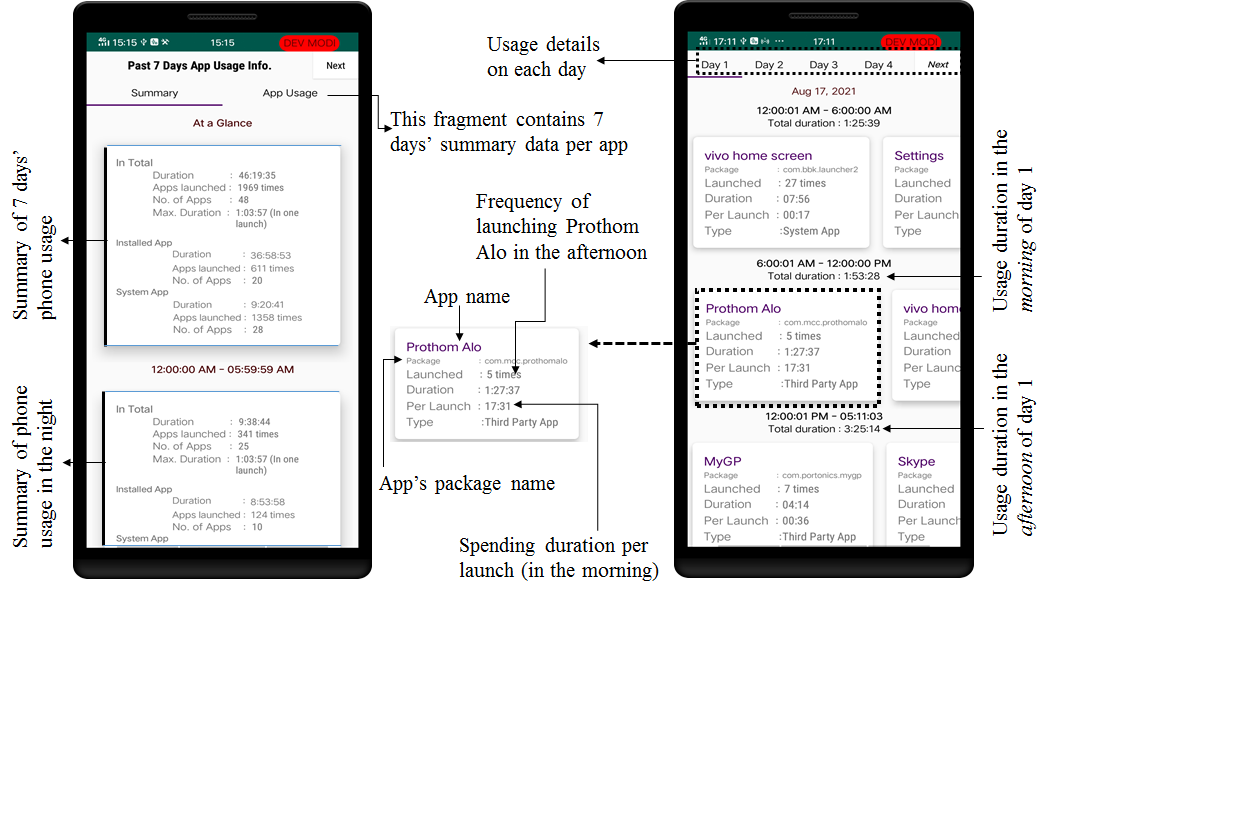


| (a) The data collection process of summary of 7 days’ app usage. |  | (b) The data collection process of app usage on a daily basis. |
| --- | --- | --- |

**Fig. A.1.** Operation of the data collection tool.

**Table A.1: Ninety-seven instances among the 10,000 instances where our app needed more than 1000 milliseconds to retrieve app usage data.**

| **Phone ID** | **Required Time in Milliseconds** |
| --- | --- |
| Phone 3 | 1048, 1132, 1273, 1322, 1368, 1436, 1511, 1530, 1535, 1600, 1697, 1966, 2641, 2724 |
| Phone 5 | 11074 |
| Phone 10 | 1346, 59188 |
| Phone 14 | 1034, 1163, 1423, 1607, 1725, 1725, 1814, 1855, 2144, 2195, 2249, 2322, 2330, 2345, 2346, 2385, 2424, 2426, 2518, 2523, 2624, 2910, 2968, 3034, 3535, 3635, 3866, 3974, 4562, 5642, 6859, 7068, 7500, 7573 |
| Phone 19 | 1013, 1088, 1117, 1206, 1667, 1808, 1825, 2019, 2767, 2800, 2831, 2881, 2897, 2925, 2938, 3087, 3525, 3650, 3683, 3690, 3808, 4740, 4807, 5534, 5574, 5613, 6245, 6364, 6450, 6541, 7037, 7053, 7114, 7493, 8893, 9303, 9726, 11183, 12344, 12995, 18422, 20321, 24104, 25864, 32293, 61087 |

# **Scale Translation**

We did the translation in the participants’ native language Bengali where 3 researchers, 2 final year B.Sc. (Bachelor of Science) students, and 4 other undergraduate students were involved. Each of the members of this group was Bangladeshi and was bilingual. In their university, the English language was the medium of study. One of the members was NIH certified and did courses on research ethics, two other researchers also did a course on research ethics. Before starting the translation, one of the researchers talked with the 4 undergraduate students to understand their difficulties in understanding the English questionnaires. Later, that researcher did the translation at first. After that, the translated questionnaire was updated and finalized based on the reviews and comments of the other members of that group.

During the scale translation, we removed the word “dead” from the 9^th^ item. There were several reasons for the removal of the word. First, the students are concerned about the “dead” word on the scale [91]. Second, the inclusion of the word “dead” may not be acceptable in all cultural contexts. For instance, very religious participants may feel uncomfortable [52]. Third, considering the higher depression rate [50] among the university students of Bangladesh, we were concerned about asking about a symptom having the word “dead”. We discussed this with a program coordinator (who is also an assistant professor of the Computer Science and Engineering (CSE) department), 1 lecturer from the CSE department, and 2 lecturers from the Department of Sociology. We found that all of the teachers are also worrying and suggested removing the word “dead”. For example, the program coordinator showed her concerns about the mental health of the depressed students after being asked a question having the word “dead”. Apart from the teachers, we also discussed with 3 more doctors from the COVID-19 hospitals who remarked that “hurting” which is also available in the 9^th^ item of the PHQ-9 scale may present the suicidal intention of a student having depression. Due to all of these and considering the mental health state amid the COVID-19 pandemic, we removed the “dead” word from the 9^th^ item.

# **App Categories and Extracted Features**

**Table C.1: Number (#) of apps, percentage of apps, and example apps for each of the 27 categories. The table is sorted by the percentage of apps a category contains.**

| **Category** | **# of Apps** | **Percentage** | **Example Apps** | **Category** | **# of Apps** | **Percentage** | **Example Apps** |
| --- | --- | --- | --- | --- | --- | --- | --- |
| Tools | 359 | 31.8 | AnyDesk, App Store | Personalization | 20 | 1.77 | Wallpapers, Themes |
| Games | 136 | 12.05 | Free Fire, Ludo Star | Finance | 15 | 1.33 | bKash, IBBL iSmart |
| Photo & Video | 94 | 8.33 | Photo Editor, YouTube | Lifestyle | 15 | 1.33 | Athan, SmartThings |
| Books & Reference | 73 | 6.47 | Al Hadith, Wikipedia | Travel & Local | 14 | 1.24 | BDTICKETS, Maps |
| Communication | 63 | 5.58 | Google Chat, Discord | Launcher Like App | 13 | 1.15 | Xperia Home, Launcher3 |
| Productivity | 62 | 5.49 | Adobe Acrobat, Calendar | Shopping | 12 | 1.06 | Daraz, Evaly |
| Entertainment | 38 | 3.37 | Mi Video, Netflix | Unknown | 11 | 0.97 | Not Applicable |
| Music & Audio | 37 | 3.28 | Radio, Spotify | Food & Drink | 10 | 0.89 | eFood, foodpanda |
| Browser & Search | 31 | 2.75 | Firefox, Google | Sports | 8 | 0.71 | OneFootball, Cricbuzz |
| Social | 31 | 2.75 | Facebook, TikTok | Weather | 8 | 0.71 | Windy, Weather |
| Education | 26 | 2.3 | Google Classroom, Zoom | News & Magazines | 7 | 0.62 | Briefing, Job Circular |
| Business | 21 | 1.86 | GoToMeeting, Teams | Medical | 4 | 0.35 | Maya, Daktarbhai |
| Health & Fitness | 20 | 1.77 | Mi Fit, Da Fit | Art & Design | 1 | 0.09 | Autodesk, SketchBook |

**Table C.2: Extracted 219 features which were used in the feature selection step. 24 hour denotes data of the whole day and 6 hour denotes data based on 6 hour intervals (Night: 12:01 AM - 6:00 AM; Morning: 6:01 AM - 12:00 PM; Afternoon: 12:01 PM - 6:00 PM; Evening: 6:01 PM - 12:00 AM).**

| **App category** | **Extracted Features** |
| --- | --- |
| Books & Reference | 'Weekday_Duration_24_Hour', 'Weekday_Launch_24_Hour', 'Weekday_#_of_Apps_24_Hour' |
| Browser & Search | 'Weekday_Duration_24_Hour', 'Weekend_Duration_24_Hour', 'Weekday_Duration_6_Hour_Mean', 'Weekday_Duration_6_Hour_SD', 'Weekend_Duration_6_Hour_Mean', 'Weekend_Duration_6_Hour_SD', 'Weekday_Entropy_24_Hour', 'Weekend_Entropy_24_Hour', 'Weekday_Launch_24_Hour', 'Weekend_Launch_24_Hour', 'Weekday_Launch_6_Hour_Mean', 'Weekday_Launch_6_Hour_SD', 'Weekend_Launch_6_Hour_Mean', 'Weekend_Launch_6_Hour_SD', 'Weekday_#_of_Apps_24_Hour', 'Weekend_#_of_Apps_24_Hour', 'Weekday_#_of_Apps_6_Hour_Mean', 'Weekday_#_of_Apps_6_Hour_SD', 'Weekend_#_of_Apps_6_Hour_Mean', 'Weekend_#_of_Apps_6_Hour_SD' |
| Communication | 'Weekday_Duration_24_Hour', 'Weekend_Duration_24_Hour', 'Weekday_Duration_6_Hour_Mean', 'Weekday_Duration_6_Hour_SD', 'Weekend_Duration_6_Hour_Mean', 'Weekend_Duration_6_Hour_SD', 'Weekday_Entropy_24_Hour', 'Weekend_Entropy_24_Hour', 'Weekday_Entropy_6_Hour_Mean', 'Weekday_Entropy_6_Hour_SD', 'Weekend_Entropy_6_Hour_Mean', 'Weekend_Entropy_6_Hour_SD', 'Weekday_Ratio_of_Hamming_24_Hour', 'Weekend_Ratio_of_Hamming_24_Hour', 'Weekday_Ratio_of_Hamming_6_Hour_Mean', 'Weekday_Ratio_of_Hamming_6_Hour_SD', 'Weekday_Launch_24_Hour', 'Weekend_Launch_24_Hour', 'Weekday_Launch_6_Hour_Mean', 'Weekday_Launch_6_Hour_SD', 'Weekend_Launch_6_Hour_Mean', 'Weekend_Launch_6_Hour_SD', 'Weekday_#_of_Apps_24_Hour', 'Weekend_#_of_Apps_24_Hour', 'Weekday_#_of_Apps_6_Hour_Mean', 'Weekday_#_of_Apps_6_Hour_SD', 'Weekend_#_of_Apps_6_Hour_Mean', 'Weekend_#_of_Apps_6_Hour_SD' |
| Education | 'Weekday_Duration_24_Hour', 'Weekday_Launch_24_Hour', 'Weekday_#_of_Apps_24_Hour' |
| Games | 'Weekday_Duration_24_Hour', 'Weekday_Launch_24_Hour', 'Weekday_#_of_Apps_24_Hour' |
| Launcher | 'Weekday__Duration_24_Hour', 'Weekend__Duration_24_Hour', 'Weekday__Duration_6_Hour_Mean', 'Weekday__Duration_6_Hour_SD', 'Weekend__Duration_6_Hour_Mean', 'Weekend__Duration_6_Hour_SD', 'Weekday__Entropy_24_Hour', 'Weekend__Entropy_24_Hour', 'Weekday__Entropy_6_Hour_Mean', 'Weekday__Entropy_6_Hour_SD', 'Weekday_Launch24_Hour', 'Weekend_Launch24_Hour', 'Weekday_Launch6_Hour_Mean', 'Weekday_Launch6_Hour_SD', 'Weekend_Launch6_Hour_Mean', 'Weekend_Launch6_Hour_SD', 'Weekday__#_of_Apps_24_Hour', 'Weekend__#_of_Apps_24_Hour', 'Weekday__#_of_Apps_6_Hour_Mean', 'Weekday__#_of_Apps_6_Hour_SD', 'Weekend__#_of_Apps_6_Hour_Mean', 'Weekend__#_of_Apps_6_Hour_SD' |
| Music & Audio | 'Weekday_Duration_24_Hour', 'Weekday_Launch_24_Hour', 'Weekday_#_of_Apps_24_Hour' |
| Photo & Video | 'Weekday_Video_Duration_24_Hour', 'Weekend_Video_Duration_24_Hour', 'Weekday_Video_Duration_6_Hour_Mean', 'Weekday_Video_Duration_6_Hour_SD', 'Weekend_Video_Duration_6_Hour_Mean', 'Weekend_Video_Duration_6_Hour_SD', 'Weekday_Video_Entropy_24_Hour', 'Weekend_Video_Entropy_24_Hour', 'Weekday_Video_Entropy_6_Hour_Mean', 'Weekday_Video_Entropy_6_Hour_SD', 'Weekday_Video_Ratio_of_Hamming_24_Hour', 'Weekend_Video_Ratio_of_Hamming_24_Hour', 'Weekday_Video_Launch_24_Hour', 'Weekend_Video_Launch_24_Hour', 'Weekday_Video_Launch_6_Hour_Mean', 'Weekday_Video_Launch_6_Hour_SD', 'Weekend_Video_Launch_6_Hour_Mean', 'Weekend_Video_Launch_6_Hour_SD', 'Weekday_Video_#_of_Apps_24_Hour', 'Weekend_Video_#_of_Apps_24_Hour', 'Weekday_Video_#_of_Apps_6_Hour_Mean', 'Weekday_Video_#_of_Apps_6_Hour_SD', 'Weekend_Video_#_of_Apps_6_Hour_Mean', 'Weekend_Video_#_of_Apps_6_Hour_SD' |
| Productivity | 'Weekday_Duration_24_Hour', 'Weekend_Duration_24_Hour', 'Weekday_Entropy_24_Hour', 'Weekend_Entropy_24_Hour', 'Weekday_Ratio_of_Hamming_24_Hour', 'Weekday_Launch_24_Hour', 'Weekend_Launch_24_Hour', 'Weekday_#_of_Apps_24_Hour', 'Weekend_#_of_Apps_24_Hour' |
| Social Media | 'Weekday_Duration_24_Hour', 'Weekend_Duration_24_Hour', 'Weekday_Duration_6_Hour_Mean', 'Weekday_Duration_6_Hour_SD', 'Weekend_Duration_6_Hour_Mean', 'Weekend_Duration_6_Hour_SD', 'Weekday_Entropy_24_Hour', 'Weekend_Entropy_24_Hour', 'Weekday_Launch_24_Hour', 'Weekend_Launch_24_Hour', 'Weekday_Launch_6_Hour_Mean', 'Weekday_Launch_6_Hour_SD', 'Weekend_Launch_6_Hour_Mean', 'Weekend_Launch_6_Hour_SD', 'Weekday_#_of_Apps_24_Hour', 'Weekend_#_of_Apps_24_Hour', 'Weekday_#_of_Apps_6_Hour_Mean', 'Weekday_#_of_Apps_6_Hour_SD', 'Weekend_#_of_Apps_6_Hour_Mean', 'Weekend_#_of_Apps_6_Hour_SD' |
| Tools | 'Weekday_Duration_24_Hour', 'Weekend_Duration_24_Hour', 'Weekday_Duration_6_Hour_Mean', 'Weekday_Duration_6_Hour_SD', 'Weekend_Duration_6_Hour_Mean', 'Weekend_Duration_6_Hour_SD', 'Weekday_Entropy_24_Hour', 'Weekend_Entropy_24_Hour', 'Weekday_Entropy_6_Hour_Mean', 'Weekday_Entropy_6_Hour_SD', 'Weekend_Entropy_6_Hour_Mean', 'Weekend_Entropy_6_Hour_SD', 'Weekday_Ratio_of_Hamming_24_Hour', 'Weekend_Ratio_of_Hamming_24_Hour', 'Weekday_Ratio_of_Hamming_6_Hour_Mean', 'Weekday_Ratio_of_Hamming_6_Hour_SD', 'Weekend_Ratio_of_Hamming_6_Hour_Mean', 'Weekend_Ratio_of_Hamming_6_Hour_SD', 'Weekday_Launch_24_Hour', 'Weekend_Launch_24_Hour', 'Weekday_Launch_6_Hour_Mean', 'Weekday_Launch_6_Hour_SD', 'Weekend_Launch_6_Hour_Mean', 'Weekend_Launch_6_Hour_SD', 'Weekday_#_of_Apps_24_Hour', 'Weekend_#_of_Apps_24_Hour', 'Weekday_#_of_Apps_6_Hour_Mean', 'Weekday_#_of_Apps_6_Hour_SD', 'Weekend_#_of_Apps_6_Hour_Mean', 'Weekend_#_of_Apps_6_Hour_SD' |
| Smartphone (Regardless of app category) | 'Weekday_Duration_24_Hour', 'Weekend_Duration_24_Hour', 'Weekday_Duration_6_Hour_Mean', 'Weekday_Duration_6_Hour_SD', 'Weekend_Duration_6_Hour_Mean', 'Weekend_Duration_6_Hour_SD', 'Weekday_Engage_Session_#_24_Hour', 'Weekend_Engage_Session_#_24_Hour', 'Weekday_Engage_Session_#_6_Hour_Mean', 'Weekday_Engage_Session_#_6_Hour_SD', 'Weekend_Engage_Session_#_6_Hour_Mean', 'Weekend_Engage_Session_#_6_Hour_SD', 'Weekday_Entropy_24_Hour', 'Weekend_Entropy_24_Hour', 'Weekday_Entropy_6_Hour_Mean', 'Weekday_Entropy_6_Hour_SD', 'Weekend_Entropy_6_Hour_Mean', 'Weekend_Entropy_6_Hour_SD', 'Weekday_Ratio_of_Hamming_24_Hour', 'Weekend_Ratio_of_Hamming_24_Hour', 'Weekday_Ratio_of_Hamming_6_Hour_Mean', 'Weekday_Ratio_of_Hamming_6_Hour_SD', 'Weekend_Ratio_of_Hamming_6_Hour_Mean', 'Weekend_Ratio_of_Hamming_6_Hour_SD', 'Weekday_Launch_24_Hour', 'Weekend_Launch_24_Hour', 'Weekday_Launch_6_Hour_Mean', 'Weekday_Launch_6_Hour_SD', 'Weekend_Launch_6_Hour_Mean', 'Weekend_Launch_6_Hour_SD', 'Weekday_Micro_Use_#_24_Hour', 'Weekend_Micro_Use_#_24_Hour', 'Weekday_Micro_Use_#_6_Hour_Mean', 'Weekday_Micro_Use_#_6_Hour_SD', 'Weekend_Micro_Use_#_6_Hour_Mean', 'Weekend_Micro_Use_#_6_Hour_SD', 'Weekday_Review_Session_#_24_Hour', 'Weekend_Review_Session_#_24_Hour', 'Weekday_Review_Session_#_6_Hour_Mean', 'Weekday_Review_Session_#_6_Hour_SD', 'Weekend_Review_Session_#_6_Hour_Mean', 'Weekend_Review_Session_#_6_Hour_SD', 'Weekday_Total_#_of_Sessions_24_Hour', 'Weekend_Total_#_of_Sessions_24_Hour', 'Weekday_Total_#_of_Sessions_6_Hour_Mean', 'Weekday_Total_#_of_Sessions_6_Hour_SD', 'Weekend_Total_#_of_Sessions_6_Hour_Mean', 'Weekend_Total_#_of_Sessions_6_Hour_SD', 'Weekday_#_of_Apps_24_Hour', 'Weekend_#_of_Apps_24_Hour', 'Weekday_#_of_Apps_6_Hour_Mean', 'Weekday_#_of_Apps_6_Hour_SD', 'Weekend_#_of_Apps_6_Hour_Mean', 'Weekend_#_of_Apps_6_Hour_SD' |

# **Model Development**

**Table D.1: The hyper-parameters which were tuned through the Bayesian Optimization method. In model development, 1234 was used as the random state. Here, max_dep=6 and n_estimators=20. choice(label, options) and uniform(label, low, high) are the functions of the hyperopt Python library [84] which was used to implement the Bayesian method. range(start, stop, step) is a built-in function of Python which was used to generate a sequence of integer numbers.**

| **Model** | **Hyper-parameters** |
| --- | --- |
| KNN | {'n_neighbors': range(1, 10)  'weights': hp.choice('weights', ['uniform', 'distance'])} |
| SVC | {'kernel': hp.choice('kernel',['rbf','poly']),  'gamma':hp.uniform('gamma', 0.0001, 1),  'C': hp.uniform('C', 0.001, 1000)} |
| Logistic Regression | {'C': hp.uniform('C', 0.001, 1000),  'solver': 'liblinear',  'class_weight': 'balanced'} |
| Multilayer Perceptron (MLP) | {'hidden_layer_sizes': hp.choice('hidden_layer_sizes', range(1, 20)),  'activation': hp.choice('activation', ['identity', 'tanh', 'logistic', 'relu']),  'max_iter': hp.choice('max_iter', range(1, 75000, 100)),  'learning_rate': hp.choice('learning_rate', ['invscaling', 'adaptive']),  'solver': hp.choice('solver', ['lbfgs'])} |
| Decision Tree | {'max_depth': hp.choice('max_depth', range(2, max_dep)),  'criterion': hp.choice('criterion', ['gini', 'entropy']),  'class_weight': hp.choice('class_weight', ['balanced'])} |
| Random Forest | {'n_estimators': hp.choice('n_estimators', range(2, n_estimators)),  'max_depth': hp.choice('max_depth', range(2, max_dep)),  'criterion': hp.choice('criterion', ['gini', 'entropy']),  'class_weight': hp.choice('class_weight', ['balanced_subsample'])} |
| CatBoost | {'iterations': hp.choice('iterations', range(2, n_estimators)),  'depth': hp.choice('depth', range(2, max_dep)),  'learning_rate': hp.uniform('learning_rate', 0.01, 1)} |
| Light GBM | {'max_depth': hp.choice('max_depth', range(2, max_dep)),  'n_estimators': hp.choice('n_estimators', range(2, n_estimators)),  'learning_rate': hp.uniform('learning_rate', 0.01, 1)} |
| AdaBoost | {'n_estimators': hp.choice('n_estimators', range(2, n_estimators)),  'learning_rate': hp.uniform('learning_rate', 0.01, 1)} |
| Gradient Boost (GB) | {'n_estimators': hp.choice('n_estimators', range(2, n_estimators)),  'max_depth': hp.choice('max_depth', range(2, max_dep)),  'learning_rate': hp.uniform('learning_rate', 0.01, 1)} |
| Extra Tree | {'n_estimators': hp.choice('n_estimators', range(2, n_estimators)),  'max_depth': hp.choice('max_depth', range(2, max_dep))} |
| XGB | {'learning_rate': hp.uniform('learning_rate', 0.01, 1),  'n_estimators': hp.choice('n_estimators', range(2, n_estimators)),  'max_depth': hp.choice('max_depth', range(2, max_dep))} |
| Naive Bayes | {} |
| Dummy | {} Dummy classifier was used as the baseline classifier. |

# **Machine Learning (ML) Models’ Performance**

**Table E.1: Performance of the ML models when selecting the features through the filter method Information Gain. GB: Gradient Boosting, KNN: K-Nearest Neighbor, MLP: Multilayer Perceptron, SVC: Support Vector Classifier.**

| **# of Features** | **Score** | **KNN** | **SVC** | **Logit** | **MLP** | **Decision Tree** | **Random Forest** | **CatBoost** | **Light GBM** | **AdaBoost** | **Gradient Boost** | **Extra Tree** | **XGBoost** | **Naive**  **Bayes** |
| --- | --- | --- | --- | --- | --- | --- | --- | --- | --- | --- | --- | --- | --- | --- |
| 5 | Sensitivity | 0.608 | 0.784 | 0.392 | 0.784 | 0.745 | 0.725 | 0.667 | 0.627 | 0.647 | 0.608 | 0.667 | 0.686 | 0.549 |
|  | Specificity | 0.653 | 0.306 | 0.571 | 0.469 | 0.592 | 0.612 | 0.612 | 0.571 | 0.612 | 0.653 | 0.408 | 0.633 | 0.367 |
|  | Precision | 0.646 | 0.541 | 0.488 | 0.606 | 0.655 | 0.661 | 0.642 | 0.604 | 0.635 | 0.646 | 0.54 | 0.66 | 0.475 |
| 6 | Sensitivity | 0.686 | 0.725 | 0.49 | 0.588 | 0.627 | 0.706 | 0.706 | 0.647 | 0.608 | 0.686 | 0.745 | 0.745 | 0.529 |
|  | Specificity | 0.592 | 0.449 | 0.571 | 0.531 | 0.714 | 0.571 | 0.714 | 0.673 | 0.592 | 0.633 | 0.408 | 0.714 | 0.429 |
|  | Precision | 0.636 | 0.578 | 0.543 | 0.566 | 0.696 | 0.632 | 0.72 | 0.673 | 0.608 | 0.66 | 0.567 | 0.731 | 0.491 |
| 7 | Sensitivity | 0.608 | 0.627 | 0.608 | 0.706 | 0.647 | 0.647 | 0.745 | 0.686 | 0.686 | 0.647 | 0.667 | 0.647 | 0.588 |
|  | Specificity | 0.612 | 0.388 | 0.551 | 0.571 | 0.755 | 0.551 | 0.714 | 0.694 | 0.653 | 0.653 | 0.367 | 0.735 | 0.469 |
|  | Precision | 0.62 | 0.516 | 0.585 | 0.632 | 0.733 | 0.6 | 0.731 | 0.7 | 0.673 | 0.66 | 0.523 | 0.717 | 0.536 |
| 8 | Sensitivity | 0.549 | 0.725 | 0.569 | 0.529 | 0.706 | 0.706 | 0.706 | 0.686 | 0.627 | 0.745 | 0.745 | 0.686 | 0.627 |
|  | Specificity | 0.429 | 0.265 | 0.531 | 0.49 | 0.755 | 0.592 | 0.612 | 0.714 | 0.612 | 0.653 | 0.51 | 0.796 | 0.469 |
|  | Precision | 0.5 | 0.507 | 0.558 | 0.519 | 0.75 | 0.643 | 0.655 | 0.714 | 0.627 | 0.691 | 0.613 | 0.778 | 0.552 |
| 9 | Sensitivity | 0.647 | 0.843 | 0.529 | 0.569 | 0.706 | 0.647 | 0.725 | 0.745 | 0.686 | 0.745 | 0.804 | 0.706 | 0.686 |
|  | Specificity | 0.49 | 0.429 | 0.49 | 0.449 | 0.776 | 0.592 | 0.633 | 0.673 | 0.735 | 0.694 | 0.51 | 0.673 | 0.388 |
|  | Precision | 0.569 | 0.606 | 0.519 | 0.518 | 0.766 | 0.623 | 0.673 | 0.704 | 0.729 | 0.717 | 0.631 | 0.692 | 0.538 |
| 10 | Sensitivity | 0.608 | 0.824 | 0.529 | 0.627 | 0.686 | 0.627 | 0.706 | 0.804 | 0.588 | 0.686 | 0.843 | 0.706 | 0.627 |
|  | Specificity | 0.449 | 0.367 | 0.469 | 0.469 | 0.776 | 0.571 | 0.551 | 0.633 | 0.612 | 0.633 | 0.449 | 0.571 | 0.347 |
|  | Precision | 0.534 | 0.575 | 0.509 | 0.552 | 0.761 | 0.604 | 0.621 | 0.695 | 0.612 | 0.66 | 0.614 | 0.632 | 0.5 |
| 11 | Sensitivity | 0.588 | 0.824 | 0.569 | 0.706 | 0.667 | 0.647 | 0.706 | 0.725 | 0.686 | 0.667 | 0.667 | 0.725 | 0.647 |
|  | Specificity | 0.388 | 0.429 | 0.49 | 0.449 | 0.714 | 0.653 | 0.571 | 0.694 | 0.612 | 0.653 | 0.388 | 0.694 | 0.347 |
|  | Precision | 0.5 | 0.6 | 0.537 | 0.571 | 0.708 | 0.66 | 0.632 | 0.712 | 0.648 | 0.667 | 0.531 | 0.712 | 0.508 |
| 12 | Sensitivity | 0.647 | 0.843 | 0.608 | 0.667 | 0.647 | 0.647 | 0.667 | 0.647 | 0.667 | 0.667 | 0.843 | 0.745 | 0.588 |
|  | Specificity | 0.469 | 0.163 | 0.449 | 0.469 | 0.796 | 0.51 | 0.571 | 0.714 | 0.673 | 0.694 | 0.449 | 0.653 | 0.367 |
|  | Precision | 0.559 | 0.512 | 0.534 | 0.567 | 0.767 | 0.579 | 0.618 | 0.702 | 0.68 | 0.694 | 0.614 | 0.691 | 0.492 |
| 13 | Sensitivity | 0.569 | 0.784 | 0.588 | 0.667 | 0.647 | 0.706 | 0.706 | 0.706 | 0.706 | 0.608 | 0.686 | 0.784 | 0.627 |
|  | Specificity | 0.429 | 0.122 | 0.51 | 0.531 | 0.755 | 0.551 | 0.633 | 0.653 | 0.673 | 0.592 | 0.408 | 0.653 | 0.388 |
|  | Precision | 0.509 | 0.482 | 0.556 | 0.596 | 0.733 | 0.621 | 0.667 | 0.679 | 0.692 | 0.608 | 0.547 | 0.702 | 0.516 |
| 14 | Sensitivity | 0.608 | 0.863 | 0.569 | 0.588 | 0.647 | 0.725 | 0.686 | 0.706 | 0.647 | 0.725 | 0.745 | 0.627 | 0.588 |
|  | Specificity | 0.388 | 0.143 | 0.571 | 0.49 | 0.755 | 0.51 | 0.633 | 0.694 | 0.653 | 0.673 | 0.347 | 0.653 | 0.367 |
|  | Precision | 0.508 | 0.512 | 0.58 | 0.545 | 0.733 | 0.607 | 0.66 | 0.706 | 0.66 | 0.698 | 0.543 | 0.653 | 0.492 |
| 15 | Sensitivity | 0.667 | 0.843 | 0.588 | 0.549 | 0.647 | 0.608 | 0.667 | 0.686 | 0.627 | 0.706 | 0.647 | 0.667 | 0.529 |
|  | Specificity | 0.469 | 0.102 | 0.551 | 0.531 | 0.776 | 0.531 | 0.551 | 0.714 | 0.694 | 0.612 | 0.388 | 0.633 | 0.388 |
|  | Precision | 0.567 | 0.494 | 0.577 | 0.549 | 0.75 | 0.574 | 0.607 | 0.714 | 0.681 | 0.655 | 0.524 | 0.654 | 0.474 |
| 16 | Sensitivity | 0.667 | 0.824 | 0.608 | 0.51 | 0.647 | 0.667 | 0.706 | 0.686 | 0.667 | 0.686 | 0.627 | 0.686 | 0.471 |
|  | Specificity | 0.449 | 0.041 | 0.49 | 0.429 | 0.694 | 0.571 | 0.633 | 0.633 | 0.531 | 0.694 | 0.367 | 0.571 | 0.388 |
|  | Precision | 0.557 | 0.472 | 0.554 | 0.481 | 0.688 | 0.618 | 0.667 | 0.66 | 0.596 | 0.7 | 0.508 | 0.625 | 0.444 |
| 17 | Sensitivity | 0.51 | 0.863 | 0.608 | 0.627 | 0.686 | 0.647 | 0.667 | 0.706 | 0.667 | 0.627 | 0.745 | 0.686 | 0.49 |
|  | Specificity | 0.408 | 0.082 | 0.51 | 0.531 | 0.796 | 0.51 | 0.551 | 0.653 | 0.673 | 0.592 | 0.367 | 0.592 | 0.367 |
|  | Precision | 0.473 | 0.494 | 0.564 | 0.582 | 0.778 | 0.579 | 0.607 | 0.679 | 0.68 | 0.615 | 0.551 | 0.636 | 0.446 |
| 18 | Sensitivity | 0.627 | 0.863 | 0.608 | 0.529 | 0.667 | 0.686 | 0.725 | 0.745 | 0.647 | 0.725 | 0.765 | 0.667 | 0.51 |
|  | Specificity | 0.388 | 0.041 | 0.49 | 0.531 | 0.735 | 0.49 | 0.551 | 0.673 | 0.633 | 0.592 | 0.327 | 0.653 | 0.367 |
|  | Precision | 0.516 | 0.484 | 0.554 | 0.54 | 0.723 | 0.583 | 0.627 | 0.704 | 0.647 | 0.649 | 0.542 | 0.667 | 0.456 |
| 19 | Sensitivity | 0.608 | 0.863 | 0.627 | 0.529 | 0.647 | 0.627 | 0.725 | 0.686 | 0.686 | 0.706 | 0.706 | 0.725 | 0.529 |
|  | Specificity | 0.469 | 0.02 | 0.551 | 0.571 | 0.714 | 0.469 | 0.592 | 0.694 | 0.571 | 0.592 | 0.469 | 0.694 | 0.327 |
|  | Precision | 0.544 | 0.478 | 0.593 | 0.562 | 0.702 | 0.552 | 0.649 | 0.7 | 0.625 | 0.643 | 0.581 | 0.712 | 0.45 |
| 20 | Sensitivity | 0.569 | 0.863 | 0.627 | 0.529 | 0.647 | 0.647 | 0.706 | 0.745 | 0.627 | 0.686 | 0.745 | 0.706 | 0.49 |
|  | Specificity | 0.327 | 0.041 | 0.571 | 0.694 | 0.755 | 0.449 | 0.51 | 0.694 | 0.673 | 0.571 | 0.531 | 0.714 | 0.347 |
|  | Precision | 0.468 | 0.484 | 0.604 | 0.643 | 0.733 | 0.55 | 0.6 | 0.717 | 0.667 | 0.625 | 0.623 | 0.72 | 0.439 |

**Table E.2: Performance of the ML models (in different depths of the base estimator Random Forest) when selecting features using the wrapper method Boruta. GB: Gradient Boosting, KNN: K-Nearest Neighbor, MLP: Multilayer Perceptron, SVC: Support Vector Classifier.**

| **Depth of the Base Estimator** | **Score** | **KNN** | **SVC** | **Logit** | **MLP** | **Decision Tree** | **Random Forest** | **CatBoost** | **Light GBM** | **AdaBoost** | **Gradient Boost** | **Extra Tree** | **XGBoost** | **Naive**  **Bayes** |
| --- | --- | --- | --- | --- | --- | --- | --- | --- | --- | --- | --- | --- | --- | --- |
| 3 | Sensitivity | 0.784 | 0.627 | 0.706 | 0.686 | 0.608 | 0.784 | 0.725 | 0.706 | 0.706 | 0.745 | 0.725 | 0.647 | 0.824 |
|  | Specificity | 0.633 | 0.612 | 0.673 | 0.633 | 0.551 | 0.633 | 0.653 | 0.633 | 0.673 | 0.673 | 0.551 | 0.673 | 0.367 |
|  | Precision | 0.69 | 0.627 | 0.692 | 0.66 | 0.585 | 0.69 | 0.685 | 0.667 | 0.692 | 0.704 | 0.627 | 0.673 | 0.575 |
| 4 | Sensitivity | 0.824 | 0.706 | 0.725 | 0.667 | 0.608 | 0.804 | 0.725 | 0.706 | 0.706 | 0.745 | 0.804 | 0.725 | 0.843 |
|  | Specificity | 0.653 | 0.633 | 0.653 | 0.592 | 0.551 | 0.612 | 0.673 | 0.653 | 0.694 | 0.653 | 0.469 | 0.653 | 0.327 |
|  | Precision | 0.712 | 0.667 | 0.685 | 0.63 | 0.585 | 0.683 | 0.698 | 0.679 | 0.706 | 0.691 | 0.612 | 0.685 | 0.566 |
| 5 | Sensitivity | 0.706 | 0.725 | 0.706 | 0.608 | 0.608 | 0.745 | 0.686 | 0.706 | 0.667 | 0.725 | 0.804 | 0.686 | 0.843 |
|  | Specificity | 0.633 | 0.612 | 0.673 | 0.592 | 0.531 | 0.571 | 0.592 | 0.571 | 0.633 | 0.633 | 0.51 | 0.633 | 0.306 |
|  | Precision | 0.667 | 0.661 | 0.692 | 0.608 | 0.574 | 0.644 | 0.636 | 0.632 | 0.654 | 0.673 | 0.631 | 0.66 | 0.558 |
| 6 | Sensitivity | 0.804 | 0.784 | 0.725 | 0.725 | 0.706 | 0.745 | 0.706 | 0.686 | 0.765 | 0.745 | 0.765 | 0.725 | 0.824 |
|  | Specificity | 0.653 | 0.571 | 0.673 | 0.571 | 0.51 | 0.653 | 0.653 | 0.714 | 0.653 | 0.714 | 0.51 | 0.714 | 0.327 |
|  | Precision | 0.707 | 0.656 | 0.698 | 0.638 | 0.6 | 0.691 | 0.679 | 0.714 | 0.696 | 0.731 | 0.619 | 0.725 | 0.56 |
| 7 | Sensitivity | 0.804 | 0.765 | 0.725 | 0.667 | 0.627 | 0.686 | 0.725 | 0.725 | 0.706 | 0.804 | 0.804 | 0.725 | 0.824 |
|  | Specificity | 0.551 | 0.571 | 0.612 | 0.551 | 0.51 | 0.612 | 0.694 | 0.531 | 0.571 | 0.653 | 0.592 | 0.673 | 0.347 |
|  | Precision | 0.651 | 0.65 | 0.661 | 0.607 | 0.571 | 0.648 | 0.712 | 0.617 | 0.632 | 0.707 | 0.672 | 0.698 | 0.568 |

**Table E.3: Performance of the ML models after selecting the features through the embedded method Random Forest. GB: Gradient Boosting, KNN: K-Nearest Neighbor, MLP: Multilayer Perceptron, SVC: Support Vector Classifier.**

| **# of Features** | **Score** | **KNN** | **SVC** | **Logit** | **MLP** | **Decision Tree** | **Random Forest** | **CatBoost** | **Light GBM** | **AdaBoost** | **Gradient Boost** | **Extra Tree** | **XGBoost** | **Naive**  **Bayes** |
| --- | --- | --- | --- | --- | --- | --- | --- | --- | --- | --- | --- | --- | --- | --- |
| 5 | Sensitivity | 0.667 | 0.627 | 0.627 | 0.667 | 0.588 | 0.686 | 0.725 | 0.686 | 0.647 | 0.627 | 0.667 | 0.608 | 0.863 |
|  | Specificity | 0.571 | 0.633 | 0.633 | 0.49 | 0.653 | 0.551 | 0.592 | 0.612 | 0.592 | 0.633 | 0.531 | 0.694 | 0.388 |
|  | Precision | 0.618 | 0.64 | 0.64 | 0.576 | 0.638 | 0.614 | 0.649 | 0.648 | 0.623 | 0.64 | 0.596 | 0.674 | 0.595 |
| 6 | Sensitivity | 0.706 | 0.569 | 0.588 | 0.627 | 0.627 | 0.745 | 0.706 | 0.667 | 0.647 | 0.686 | 0.647 | 0.706 | 0.784 |
|  | Specificity | 0.551 | 0.531 | 0.694 | 0.653 | 0.714 | 0.571 | 0.571 | 0.592 | 0.612 | 0.633 | 0.51 | 0.653 | 0.449 |
|  | Precision | 0.621 | 0.558 | 0.667 | 0.653 | 0.696 | 0.644 | 0.632 | 0.63 | 0.635 | 0.66 | 0.579 | 0.679 | 0.597 |
| 7 | Sensitivity | 0.627 | 0.667 | 0.706 | 0.667 | 0.706 | 0.765 | 0.706 | 0.706 | 0.588 | 0.804 | 0.745 | 0.686 | 0.784 |
|  | Specificity | 0.551 | 0.592 | 0.673 | 0.571 | 0.653 | 0.612 | 0.551 | 0.571 | 0.755 | 0.653 | 0.449 | 0.592 | 0.469 |
|  | Precision | 0.593 | 0.63 | 0.692 | 0.618 | 0.679 | 0.672 | 0.621 | 0.632 | 0.714 | 0.707 | 0.585 | 0.636 | 0.606 |
| 8 | Sensitivity | 0.667 | 0.627 | 0.647 | 0.549 | 0.706 | 0.725 | 0.667 | 0.706 | 0.667 | 0.667 | 0.706 | 0.667 | 0.745 |
|  | Specificity | 0.673 | 0.673 | 0.694 | 0.653 | 0.735 | 0.551 | 0.571 | 0.673 | 0.633 | 0.633 | 0.51 | 0.592 | 0.49 |
|  | Precision | 0.68 | 0.667 | 0.688 | 0.622 | 0.735 | 0.627 | 0.618 | 0.692 | 0.654 | 0.654 | 0.6 | 0.63 | 0.603 |
| 9 | Sensitivity | 0.627 | 0.588 | 0.686 | 0.608 | 0.686 | 0.627 | 0.765 | 0.745 | 0.647 | 0.706 | 0.706 | 0.706 | 0.706 |
|  | Specificity | 0.571 | 0.571 | 0.673 | 0.592 | 0.755 | 0.592 | 0.592 | 0.714 | 0.776 | 0.673 | 0.51 | 0.714 | 0.51 |
|  | Precision | 0.604 | 0.588 | 0.686 | 0.608 | 0.745 | 0.615 | 0.661 | 0.731 | 0.75 | 0.692 | 0.6 | 0.72 | 0.6 |
| 10 | Sensitivity | 0.588 | 0.667 | 0.686 | 0.706 | 0.667 | 0.686 | 0.627 | 0.745 | 0.608 | 0.706 | 0.608 | 0.686 | 0.706 |
|  | Specificity | 0.633 | 0.551 | 0.673 | 0.531 | 0.735 | 0.653 | 0.673 | 0.714 | 0.735 | 0.633 | 0.531 | 0.653 | 0.531 |
|  | Precision | 0.625 | 0.607 | 0.686 | 0.61 | 0.723 | 0.673 | 0.667 | 0.731 | 0.705 | 0.667 | 0.574 | 0.673 | 0.61 |
| 11 | Sensitivity | 0.627 | 0.627 | 0.686 | 0.686 | 0.706 | 0.569 | 0.667 | 0.725 | 0.549 | 0.725 | 0.725 | 0.725 | 0.706 |
|  | Specificity | 0.673 | 0.51 | 0.714 | 0.51 | 0.714 | 0.633 | 0.633 | 0.755 | 0.735 | 0.694 | 0.531 | 0.714 | 0.592 |
|  | Precision | 0.667 | 0.571 | 0.714 | 0.593 | 0.72 | 0.617 | 0.654 | 0.755 | 0.683 | 0.712 | 0.617 | 0.725 | 0.643 |
| 12 | Sensitivity | 0.706 | 0.667 | 0.706 | 0.706 | 0.686 | 0.627 | 0.667 | 0.725 | 0.569 | 0.804 | 0.686 | 0.725 | 0.686 |
|  | Specificity | 0.694 | 0.551 | 0.755 | 0.612 | 0.735 | 0.592 | 0.714 | 0.755 | 0.673 | 0.694 | 0.571 | 0.755 | 0.612 |
|  | Precision | 0.706 | 0.607 | 0.75 | 0.655 | 0.729 | 0.615 | 0.708 | 0.755 | 0.644 | 0.732 | 0.625 | 0.755 | 0.648 |
| 13 | Sensitivity | 0.667 | 0.745 | 0.647 | 0.647 | 0.706 | 0.608 | 0.745 | 0.706 | 0.647 | 0.686 | 0.667 | 0.647 | 0.706 |
|  | Specificity | 0.714 | 0.469 | 0.673 | 0.531 | 0.735 | 0.673 | 0.612 | 0.694 | 0.735 | 0.633 | 0.51 | 0.694 | 0.612 |
|  | Precision | 0.708 | 0.594 | 0.673 | 0.589 | 0.735 | 0.66 | 0.667 | 0.706 | 0.717 | 0.66 | 0.586 | 0.688 | 0.655 |
| 14 | Sensitivity | 0.706 | 0.667 | 0.686 | 0.647 | 0.686 | 0.725 | 0.647 | 0.667 | 0.608 | 0.725 | 0.686 | 0.725 | 0.647 |
|  | Specificity | 0.714 | 0.449 | 0.673 | 0.633 | 0.714 | 0.592 | 0.653 | 0.633 | 0.673 | 0.694 | 0.49 | 0.633 | 0.592 |
|  | Precision | 0.72 | 0.557 | 0.686 | 0.647 | 0.714 | 0.649 | 0.66 | 0.654 | 0.66 | 0.712 | 0.583 | 0.673 | 0.623 |
| 15 | Sensitivity | 0.745 | 0.686 | 0.647 | 0.647 | 0.667 | 0.608 | 0.706 | 0.725 | 0.745 | 0.784 | 0.667 | 0.725 | 0.627 |
|  | Specificity | 0.612 | 0.367 | 0.673 | 0.551 | 0.653 | 0.633 | 0.551 | 0.612 | 0.694 | 0.714 | 0.469 | 0.612 | 0.633 |
|  | Precision | 0.667 | 0.53 | 0.673 | 0.6 | 0.667 | 0.633 | 0.621 | 0.661 | 0.717 | 0.741 | 0.567 | 0.661 | 0.64 |
| 16 | Sensitivity | 0.588 | 0.647 | 0.647 | 0.588 | 0.706 | 0.627 | 0.706 | 0.725 | 0.529 | 0.686 | 0.608 | 0.647 | 0.608 |
|  | Specificity | 0.571 | 0.429 | 0.694 | 0.49 | 0.653 | 0.612 | 0.633 | 0.694 | 0.653 | 0.592 | 0.551 | 0.592 | 0.653 |
|  | Precision | 0.588 | 0.541 | 0.688 | 0.545 | 0.679 | 0.627 | 0.667 | 0.712 | 0.614 | 0.636 | 0.585 | 0.623 | 0.646 |
| 17 | Sensitivity | 0.588 | 0.667 | 0.569 | 0.608 | 0.667 | 0.765 | 0.529 | 0.647 | 0.608 | 0.667 | 0.627 | 0.686 | 0.608 |
|  | Specificity | 0.612 | 0.367 | 0.694 | 0.49 | 0.592 | 0.653 | 0.592 | 0.673 | 0.735 | 0.571 | 0.469 | 0.551 | 0.673 |
|  | Precision | 0.612 | 0.523 | 0.659 | 0.554 | 0.63 | 0.696 | 0.574 | 0.673 | 0.705 | 0.618 | 0.552 | 0.614 | 0.66 |
| 18 | Sensitivity | 0.608 | 0.667 | 0.588 | 0.608 | 0.686 | 0.706 | 0.706 | 0.627 | 0.588 | 0.667 | 0.667 | 0.627 | 0.588 |
|  | Specificity | 0.612 | 0.286 | 0.633 | 0.653 | 0.633 | 0.531 | 0.551 | 0.714 | 0.673 | 0.571 | 0.551 | 0.673 | 0.673 |
|  | Precision | 0.62 | 0.493 | 0.625 | 0.646 | 0.66 | 0.61 | 0.621 | 0.696 | 0.652 | 0.618 | 0.607 | 0.667 | 0.652 |
| 19 | Sensitivity | 0.529 | 0.725 | 0.549 | 0.529 | 0.667 | 0.686 | 0.706 | 0.686 | 0.588 | 0.706 | 0.627 | 0.686 | 0.569 |
|  | Specificity | 0.531 | 0.265 | 0.633 | 0.49 | 0.653 | 0.612 | 0.49 | 0.735 | 0.673 | 0.592 | 0.449 | 0.653 | 0.694 |
|  | Precision | 0.54 | 0.507 | 0.609 | 0.519 | 0.667 | 0.648 | 0.59 | 0.729 | 0.652 | 0.643 | 0.542 | 0.673 | 0.659 |
| 20 | Sensitivity | 0.569 | 0.706 | 0.549 | 0.647 | 0.647 | 0.647 | 0.647 | 0.627 | 0.588 | 0.725 | 0.627 | 0.706 | 0.549 |
|  | Specificity | 0.531 | 0.429 | 0.673 | 0.612 | 0.653 | 0.551 | 0.612 | 0.571 | 0.714 | 0.694 | 0.592 | 0.673 | 0.673 |
|  | Precision | 0.558 | 0.562 | 0.636 | 0.635 | 0.66 | 0.6 | 0.635 | 0.604 | 0.682 | 0.712 | 0.615 | 0.692 | 0.636 |

**Table E.4: ML models performance after selecting features in different thresholds of the Stable feature selection approach. Here, the threshold denotes the minimum percentage of the 1000 bootstrapped subsamples that contain a particular feature to be selected. GB: Gradient Boosting, KNN: K-Nearest Neighbor, MLP: Multilayer Perceptron, SVC: Support Vector Classifier.**

| **Threshold** | **Score** | **KNN** | **SVC** | **Logit** | **MLP** | **Decision Tree** | **Random Forest** | **CatBoost** | **Light GBM** | **AdaBoost** | **Gradient Boost** | **Extra Tree** | **XGBoost** | **Naive**  **Bayes** | |
| --- | --- | --- | --- | --- | --- | --- | --- | --- | --- | --- | --- | --- | --- | --- | --- |
| 0.5 | Sensitivity | 0.51 | 0.51 | 0.49 | 0.471 | 0.627 | 0.706 | 0.627 | 0.686 | 0.725 | 0.588 | 0.431 | 0.627 | 0.314 | |
|  | Specificity | 0.49 | 0.204 | 0.551 | 0.551 | 0.633 | 0.592 | 0.51 | 0.673 | 0.714 | 0.51 | 0.51 | 0.694 | 0.571 | |
|  | Precision | 0.51 | 0.4 | 0.532 | 0.522 | 0.64 | 0.643 | 0.571 | 0.686 | 0.725 | 0.556 | 0.478 | 0.681 | 0.432 | |
| 0.51 | Sensitivity | 0.49 | 0.471 | 0.471 | 0.49 | 0.647 | 0.529 | 0.647 | 0.667 | 0.745 | 0.588 | 0.49 | 0.706 | 0.373 | |
|  | Specificity | 0.429 | 0.367 | 0.551 | 0.571 | 0.612 | 0.633 | 0.408 | 0.571 | 0.673 | 0.51 | 0.408 | 0.592 | 0.551 | |
|  | Precision | 0.472 | 0.436 | 0.522 | 0.543 | 0.635 | 0.6 | 0.532 | 0.618 | 0.704 | 0.556 | 0.463 | 0.643 | 0.463 | |
| 0.52 | Sensitivity | 0.49 | 0.471 | 0.529 | 0.451 | 0.588 | 0.667 | 0.647 | 0.608 | 0.725 | 0.569 | 0.549 | 0.608 | 0.373 | |
|  | Specificity | 0.429 | 0.449 | 0.571 | 0.571 | 0.673 | 0.592 | 0.592 | 0.571 | 0.735 | 0.531 | 0.449 | 0.592 | 0.551 | |
|  | Precision | 0.472 | 0.471 | 0.562 | 0.523 | 0.652 | 0.63 | 0.623 | 0.596 | 0.74 | 0.558 | 0.509 | 0.608 | 0.463 | |
| 0.53 | Sensitivity | 0.471 | 0.49 | 0.529 | 0.49 | 0.608 | 0.608 | 0.49 | 0.549 | 0.725 | 0.608 | 0.667 | 0.627 | 0.353 | |
|  | Specificity | 0.469 | 0.327 | 0.51 | 0.571 | 0.653 | 0.551 | 0.592 | 0.551 | 0.714 | 0.571 | 0.367 | 0.571 | 0.571 | |
|  | Precision | 0.48 | 0.431 | 0.529 | 0.543 | 0.646 | 0.585 | 0.556 | 0.56 | 0.725 | 0.596 | 0.523 | 0.604 | 0.462 | |
| 0.54 | Sensitivity | 0.51 | 0.451 | 0.431 | 0.412 | 0.608 | 0.667 | 0.627 | 0.569 | 0.706 | 0.588 | 0.569 | 0.608 | 0.392 | |
|  | Specificity | 0.51 | 0.388 | 0.531 | 0.551 | 0.653 | 0.612 | 0.612 | 0.571 | 0.755 | 0.633 | 0.429 | 0.633 | 0.551 | |
|  | Precision | 0.52 | 0.434 | 0.489 | 0.488 | 0.646 | 0.642 | 0.627 | 0.58 | 0.75 | 0.625 | 0.509 | 0.633 | 0.476 | |
| 0.55 | Sensitivity | 0.471 | 0.471 | 0.412 | 0.471 | 0.627 | 0.686 | 0.647 | 0.588 | 0.725 | 0.608 | 0.725 | 0.647 | 0.333 | |
|  | Specificity | 0.408 | 0.408 | 0.633 | 0.571 | 0.673 | 0.571 | 0.612 | 0.633 | 0.735 | 0.612 | 0.551 | 0.571 | 0.531 | |
|  | Precision | 0.453 | 0.453 | 0.538 | 0.533 | 0.667 | 0.625 | 0.635 | 0.625 | 0.74 | 0.62 | 0.627 | 0.611 | 0.425 | |
| 0.56 | Sensitivity | 0.451 | 0.471 | 0.412 | 0.431 | 0.647 | 0.608 | 0.569 | 0.647 | 0.725 | 0.706 | 0.647 | 0.686 | 0.373 | |
|  | Specificity | 0.51 | 0.429 | 0.612 | 0.551 | 0.694 | 0.633 | 0.51 | 0.592 | 0.735 | 0.673 | 0.49 | 0.612 | 0.51 | |
|  | Precision | 0.489 | 0.462 | 0.525 | 0.5 | 0.688 | 0.633 | 0.547 | 0.623 | 0.74 | 0.692 | 0.569 | 0.648 | 0.442 | |
| 0.57 | Sensitivity | 0.49 | 0.451 | 0.471 | 0.549 | 0.647 | 0.627 | 0.608 | 0.667 | 0.745 | 0.667 | 0.667 | 0.686 | 0.412 | |
|  | Specificity | 0.49 | 0.347 | 0.571 | 0.551 | 0.714 | 0.571 | 0.592 | 0.592 | 0.755 | 0.571 | 0.429 | 0.653 | 0.531 | |
|  | Precision | 0.5 | 0.418 | 0.533 | 0.56 | 0.702 | 0.604 | 0.608 | 0.63 | 0.76 | 0.618 | 0.548 | 0.673 | 0.477 | |
| 0.58 | Sensitivity | 0.51 | 0.588 | 0.529 | 0.529 | 0.627 | 0.725 | 0.608 | 0.608 | 0.686 | 0.706 | 0.588 | 0.627 | 0.49 | |
|  | Specificity | 0.49 | 0.245 | 0.551 | 0.592 | 0.673 | 0.571 | 0.571 | 0.653 | 0.755 | 0.612 | 0.408 | 0.714 | 0.49 | |
|  | Precision | 0.51 | 0.448 | 0.551 | 0.574 | 0.667 | 0.638 | 0.596 | 0.646 | 0.745 | 0.655 | 0.508 | 0.696 | 0.5 | |
| 0.59 | Sensitivity | 0.588 | 0.608 | 0.51 | 0.451 | 0.627 | 0.627 | 0.647 | 0.647 | 0.745 | 0.686 | 0.529 | 0.627 | 0.471 | |
|  | Specificity | 0.551 | 0.367 | 0.592 | 0.551 | 0.694 | 0.673 | 0.633 | 0.673 | 0.714 | 0.653 | 0.469 | 0.673 | 0.51 | |
|  | Precision | 0.577 | 0.5 | 0.565 | 0.511 | 0.681 | 0.667 | 0.647 | 0.673 | 0.731 | 0.673 | 0.509 | 0.667 | 0.5 | |
| 0.6 | Sensitivity | 0.588 | 0.529 | 0.529 | 0.569 | 0.608 | 0.608 | 0.569 | 0.647 | 0.725 | 0.647 | 0.608 | 0.627 | 0.49 | |
|  | Specificity | 0.51 | 0.408 | 0.571 | 0.571 | 0.694 | 0.592 | 0.551 | 0.653 | 0.735 | 0.653 | 0.51 | 0.633 | 0.469 | |
|  | Precision | 0.556 | 0.482 | 0.562 | 0.58 | 0.674 | 0.608 | 0.569 | 0.66 | 0.74 | 0.66 | 0.564 | 0.64 | 0.49 | |
| 0.61 | Sensitivity | 0.608 | 0.549 | 0.588 | 0.569 | 0.608 | 0.627 | 0.667 | 0.608 | 0.686 | 0.686 | 0.667 | 0.667 | 0.51 | |
|  | Specificity | 0.531 | 0.49 | 0.592 | 0.571 | 0.694 | 0.551 | 0.551 | 0.694 | 0.714 | 0.694 | 0.367 | 0.633 | 0.49 | |
|  | Precision | 0.574 | 0.528 | 0.6 | 0.58 | 0.674 | 0.593 | 0.607 | 0.674 | 0.714 | 0.7 | 0.523 | 0.654 | 0.51 | |
| 0.62 | Sensitivity | 0.647 | 0.608 | 0.569 | 0.588 | 0.647 | 0.745 | 0.647 | 0.686 | 0.647 | 0.667 | 0.608 | 0.647 | 0.529 | |
|  | Specificity | 0.551 | 0.347 | 0.592 | 0.531 | 0.694 | 0.612 | 0.571 | 0.673 | 0.735 | 0.633 | 0.449 | 0.612 | 0.49 | |
|  | Precision | 0.6 | 0.492 | 0.592 | 0.566 | 0.688 | 0.667 | 0.611 | 0.686 | 0.717 | 0.654 | 0.534 | 0.635 | 0.519 | |
| 0.63 | Sensitivity | 0.647 | 0.627 | 0.588 | 0.549 | 0.667 | 0.745 | 0.588 | 0.686 | 0.706 | 0.686 | 0.627 | 0.647 | 0.588 | |
|  | Specificity | 0.592 | 0.469 | 0.551 | 0.551 | 0.714 | 0.551 | 0.592 | 0.673 | 0.755 | 0.571 | 0.49 | 0.592 | 0.51 | |
|  | Precision | 0.623 | 0.552 | 0.577 | 0.56 | 0.708 | 0.633 | 0.6 | 0.686 | 0.75 | 0.625 | 0.561 | 0.623 | 0.556 | |
| 0.64 | Sensitivity | 0.686 | 0.627 | 0.608 | 0.529 | 0.667 | 0.549 | 0.569 | 0.706 | 0.647 | 0.706 | 0.667 | 0.667 | 0.588 | |
|  | Specificity | 0.653 | 0.469 | 0.633 | 0.551 | 0.735 | 0.51 | 0.592 | 0.714 | 0.694 | 0.653 | 0.49 | 0.633 | 0.551 | |
|  | Precision | 0.673 | 0.552 | 0.633 | 0.551 | 0.723 | 0.538 | 0.592 | 0.72 | 0.688 | 0.679 | 0.576 | 0.654 | 0.577 | |
| 0.65 | Sensitivity | 0.667 | 0.745 | 0.627 | 0.608 | 0.608 | 0.627 | 0.588 | 0.765 | 0.647 | 0.667 | 0.647 | 0.706 | 0.588 | |
|  | Specificity | 0.592 | 0.49 | 0.612 | 0.49 | 0.735 | 0.531 | 0.633 | 0.776 | 0.633 | 0.653 | 0.429 | 0.612 | 0.531 | |
|  | Precision | 0.63 | 0.603 | 0.627 | 0.554 | 0.705 | 0.582 | 0.625 | 0.78 | 0.647 | 0.667 | 0.541 | 0.655 | 0.566 | |
| 0.66 | Sensitivity | 0.647 | 0.745 | 0.608 | 0.529 | 0.627 | 0.627 | 0.588 | 0.745 | 0.588 | 0.706 | 0.686 | 0.647 | 0.608 | |
|  | Specificity | 0.551 | 0.469 | 0.592 | 0.551 | 0.673 | 0.612 | 0.612 | 0.776 | 0.673 | 0.653 | 0.429 | 0.571 | 0.531 | |
|  | Precision | 0.6 | 0.594 | 0.608 | 0.551 | 0.667 | 0.627 | 0.612 | 0.776 | 0.652 | 0.679 | 0.556 | 0.611 | 0.574 | |
| 0.67 | Sensitivity | 0.686 | 0.725 | 0.608 | 0.569 | 0.686 | 0.647 | 0.588 | 0.725 | 0.588 | 0.725 | 0.784 | 0.627 | 0.647 | |
|  | Specificity | 0.531 | 0.469 | 0.592 | 0.531 | 0.694 | 0.551 | 0.653 | 0.714 | 0.714 | 0.612 | 0.408 | 0.531 | 0.49 | |
|  | Precision | 0.603 | 0.587 | 0.608 | 0.558 | 0.7 | 0.6 | 0.638 | 0.725 | 0.682 | 0.661 | 0.58 | 0.582 | 0.569 | |
| 0.68 | Sensitivity | 0.686 | 0.745 | 0.608 | 0.549 | 0.647 | 0.608 | 0.706 | 0.725 | 0.608 | 0.725 | 0.725 | 0.667 | 0.627 | |
|  | Specificity | 0.571 | 0.531 | 0.551 | 0.551 | 0.694 | 0.633 | 0.653 | 0.673 | 0.673 | 0.612 | 0.429 | 0.571 | 0.469 | |
|  | Precision | 0.625 | 0.623 | 0.585 | 0.56 | 0.688 | 0.633 | 0.679 | 0.698 | 0.66 | 0.661 | 0.569 | 0.618 | 0.552 | |
| 0.69 | Sensitivity | 0.667 | 0.706 | 0.627 | 0.588 | 0.686 | 0.667 | 0.667 | 0.647 | 0.608 | 0.686 | 0.765 | 0.667 | 0.647 | |
|  | Specificity | 0.449 | 0.531 | 0.571 | 0.551 | 0.694 | 0.592 | 0.612 | 0.694 | 0.694 | 0.571 | 0.347 | 0.633 | 0.429 | |
|  | Precision | 0.557 | 0.61 | 0.604 | 0.577 | 0.7 | 0.63 | 0.642 | 0.688 | 0.674 | 0.625 | 0.549 | 0.654 | 0.541 | |
| 0.7 | Sensitivity | 0.667 | 0.765 | 0.608 | 0.569 | 0.647 | 0.686 | 0.647 | 0.667 | 0.588 | 0.706 | 0.765 | 0.647 | 0.686 | |
|  | Specificity | 0.551 | 0.429 | 0.551 | 0.531 | 0.694 | 0.612 | 0.592 | 0.673 | 0.735 | 0.551 | 0.429 | 0.653 | 0.429 | |
|  | Precision | 0.607 | 0.582 | 0.585 | 0.558 | 0.688 | 0.648 | 0.623 | 0.68 | 0.698 | 0.621 | 0.582 | 0.66 | 0.556 | |
| 0.71 | Sensitivity | 0.706 | 0.745 | 0.647 | 0.608 | 0.686 | 0.647 | 0.667 | 0.765 | 0.667 | 0.765 | 0.804 | 0.667 | 0.706 | |
|  | Specificity | 0.51 | 0.51 | 0.571 | 0.531 | 0.653 | 0.633 | 0.551 | 0.755 | 0.694 | 0.592 | 0.429 | 0.633 | 0.408 | |
|  | Precision | 0.6 | 0.613 | 0.611 | 0.574 | 0.673 | 0.647 | 0.607 | 0.765 | 0.694 | 0.661 | 0.594 | 0.654 | 0.554 | |
| 0.72 | Sensitivity | 0.765 | 0.706 | 0.627 | 0.588 | 0.667 | 0.706 | 0.686 | 0.725 | 0.667 | 0.745 | 0.667 | 0.647 | 0.745 | |
|  | Specificity | 0.51 | 0.449 | 0.571 | 0.612 | 0.673 | 0.571 | 0.551 | 0.735 | 0.673 | 0.612 | 0.429 | 0.633 | 0.388 | |
|  | Precision | 0.619 | 0.571 | 0.604 | 0.612 | 0.68 | 0.632 | 0.614 | 0.74 | 0.68 | 0.667 | 0.548 | 0.647 | 0.559 | |
| 0.73 | Sensitivity | 0.765 | 0.745 | 0.627 | 0.706 | 0.706 | 0.667 | 0.706 | 0.745 | 0.627 | 0.745 | 0.706 | 0.667 | 0.725 | |
|  | Specificity | 0.469 | 0.51 | 0.571 | 0.571 | 0.653 | 0.612 | 0.531 | 0.714 | 0.673 | 0.653 | 0.449 | 0.653 | 0.388 | |
|  | Precision | 0.6 | 0.613 | 0.604 | 0.632 | 0.679 | 0.642 | 0.61 | 0.731 | 0.667 | 0.691 | 0.571 | 0.667 | 0.552 | |
| 0.74 | Sensitivity | 0.784 | 0.765 | 0.686 | 0.725 | 0.667 | 0.745 | 0.667 | 0.765 | 0.627 | 0.706 | 0.706 | 0.686 | 0.784 | |
|  | Specificity | 0.49 | 0.429 | 0.571 | 0.531 | 0.673 | 0.592 | 0.633 | 0.735 | 0.673 | 0.673 | 0.51 | 0.673 | 0.408 | |
|  | Precision | 0.615 | 0.582 | 0.625 | 0.617 | 0.68 | 0.655 | 0.654 | 0.75 | 0.667 | 0.692 | 0.6 | 0.686 | 0.58 | |
| 0.75 | Sensitivity | 0.745 | 0.784 | 0.706 | 0.627 | 0.667 | 0.725 | 0.706 | 0.765 | 0.608 | 0.725 | 0.647 | 0.706 | 0.706 | |
|  | Specificity | 0.429 | 0.449 | 0.612 | 0.531 | 0.673 | 0.571 | 0.612 | 0.735 | 0.653 | 0.653 | 0.449 | 0.694 | 0.408 | |
|  | Precision | 0.576 | 0.597 | 0.655 | 0.582 | 0.68 | 0.638 | 0.655 | 0.75 | 0.646 | 0.685 | 0.55 | 0.706 | 0.554 | |
| 0.76 | Sensitivity | 0.765 | 0.824 | 0.706 | 0.627 | 0.686 | 0.706 | 0.765 | 0.745 | 0.706 | 0.745 | 0.686 | 0.765 | 0.706 | |
|  | Specificity | 0.469 | 0.388 | 0.612 | 0.51 | 0.694 | 0.551 | 0.653 | 0.735 | 0.612 | 0.633 | 0.429 | 0.735 | 0.429 | |
|  | Precision | 0.6 | 0.583 | 0.655 | 0.571 | 0.7 | 0.621 | 0.696 | 0.745 | 0.655 | 0.679 | 0.556 | 0.75 | 0.562 | |
| 0.77 | Sensitivity | 0.745 | 0.843 | 0.686 | 0.686 | 0.706 | 0.784 | 0.745 | 0.824 | 0.725 | 0.706 | 0.647 | 0.745 | 0.725 | |
|  | Specificity | 0.469 | 0.429 | 0.633 | 0.469 | 0.694 | 0.612 | 0.653 | 0.714 | 0.633 | 0.694 | 0.388 | 0.735 | 0.469 | |
|  | Precision | 0.594 | 0.606 | 0.66 | 0.574 | 0.706 | 0.678 | 0.691 | 0.75 | 0.673 | 0.706 | 0.524 | 0.745 | 0.587 | |
| 0.78 | Sensitivity | 0.745 | 0.784 | 0.686 | 0.627 | 0.706 | 0.765 | 0.706 | 0.843 | 0.706 | 0.686 | 0.686 | 0.765 | 0.706 | |
|  | Specificity | 0.408 | 0.408 | 0.612 | 0.429 | 0.673 | 0.592 | 0.633 | 0.694 | 0.714 | 0.653 | 0.388 | 0.673 | 0.429 | |
|  | Precision | 0.567 | 0.58 | 0.648 | 0.533 | 0.692 | 0.661 | 0.667 | 0.741 | 0.72 | 0.673 | 0.538 | 0.709 | 0.562 | |
| 0.79 | Sensitivity | 0.706 | 0.706 | 0.706 | 0.647 | 0.667 | 0.706 | 0.647 | 0.824 | 0.706 | 0.667 | 0.627 | 0.725 | 0.706 | |
|  | Specificity | 0.388 | 0.408 | 0.612 | 0.429 | 0.694 | 0.633 | 0.612 | 0.694 | 0.673 | 0.673 | 0.429 | 0.673 | 0.449 | |
|  | Precision | 0.545 | 0.554 | 0.655 | 0.541 | 0.694 | 0.667 | 0.635 | 0.737 | 0.692 | 0.68 | 0.533 | 0.698 | 0.571 | |
| 0.8 | Sensitivity | 0.706 | 0.686 | 0.706 | 0.686 | 0.647 | 0.706 | 0.667 | 0.784 | 0.706 | 0.706 | 0.647 | 0.686 | 0.706 | |
|  | Specificity | 0.408 | 0.449 | 0.592 | 0.429 | 0.694 | 0.653 | 0.592 | 0.653 | 0.592 | 0.653 | 0.49 | 0.673 | 0.429 | |
|  | Precision | 0.554 | 0.565 | 0.643 | 0.556 | 0.688 | 0.679 | 0.63 | 0.702 | 0.643 | 0.679 | 0.569 | 0.686 | 0.562 | |
| 0.81 | Sensitivity | 0.745 | 0.686 | 0.667 | 0.667 | 0.647 | 0.647 | 0.647 | 0.725 | 0.667 | 0.686 | 0.686 | 0.627 | 0.706 | |
|  | Specificity | 0.49 | 0.388 | 0.592 | 0.51 | 0.673 | 0.592 | 0.592 | 0.653 | 0.571 | 0.612 | 0.469 | 0.633 | 0.408 | |
|  | Precision | 0.603 | 0.538 | 0.63 | 0.586 | 0.673 | 0.623 | 0.623 | 0.685 | 0.618 | 0.648 | 0.574 | 0.64 | 0.554 | |
| 0.82 | Sensitivity | 0.725 | 0.647 | 0.667 | 0.647 | 0.647 | 0.627 | 0.588 | 0.784 | 0.627 | 0.627 | 0.667 | 0.569 | 0.706 | |
|  | Specificity | 0.469 | 0.429 | 0.592 | 0.449 | 0.592 | 0.633 | 0.531 | 0.633 | 0.551 | 0.592 | 0.469 | 0.612 | 0.449 | |
|  | Precision | 0.587 | 0.541 | 0.63 | 0.55 | 0.623 | 0.64 | 0.566 | 0.69 | 0.593 | 0.615 | 0.567 | 0.604 | 0.571 | |
| 0.83 | Sensitivity | 0.627 | 0.706 | 0.686 | 0.549 | 0.667 | 0.745 | 0.627 | 0.765 | 0.627 | 0.706 | 0.706 | 0.667 | 0.706 | |
|  | Specificity | 0.347 | 0.429 | 0.571 | 0.429 | 0.612 | 0.653 | 0.551 | 0.633 | 0.653 | 0.653 | 0.49 | 0.612 | 0.429 | |
|  | Precision | 0.5 | 0.562 | 0.625 | 0.5 | 0.642 | 0.691 | 0.593 | 0.684 | 0.653 | 0.679 | 0.59 | 0.642 | 0.562 | |
| 0.84 | Sensitivity | 0.686 | 0.667 | 0.647 | 0.569 | 0.667 | 0.686 | 0.627 | 0.745 | 0.608 | 0.706 | 0.686 | 0.686 | 0.667 | |
|  | Specificity | 0.51 | 0.408 | 0.592 | 0.469 | 0.612 | 0.653 | 0.531 | 0.673 | 0.612 | 0.571 | 0.429 | 0.633 | 0.469 | |
|  | Precision | 0.593 | 0.54 | 0.623 | 0.527 | 0.642 | 0.673 | 0.582 | 0.704 | 0.62 | 0.632 | 0.556 | 0.66 | 0.567 | |
| 0.85 | Sensitivity | 0.667 | 0.686 | 0.647 | 0.588 | 0.608 | 0.627 | 0.647 | 0.745 | 0.627 | 0.686 | 0.745 | 0.667 | 0.706 | |
|  | Specificity | 0.551 | 0.531 | 0.612 | 0.633 | 0.592 | 0.694 | 0.612 | 0.755 | 0.653 | 0.673 | 0.429 | 0.653 | 0.469 | |
|  | Precision | 0.607 | 0.603 | 0.635 | 0.625 | 0.608 | 0.681 | 0.635 | 0.76 | 0.653 | 0.686 | 0.576 | 0.667 | 0.581 | |
| 0.86 | Sensitivity | 0.745 | 0.627 | 0.627 | 0.529 | 0.608 | 0.588 | 0.667 | 0.667 | 0.647 | 0.667 | 0.627 | 0.686 | 0.627 | |
|  | Specificity | 0.612 | 0.633 | 0.633 | 0.694 | 0.633 | 0.714 | 0.653 | 0.796 | 0.694 | 0.694 | 0.49 | 0.673 | 0.531 | |
|  | Precision | 0.667 | 0.64 | 0.64 | 0.643 | 0.633 | 0.682 | 0.667 | 0.773 | 0.688 | 0.694 | 0.561 | 0.686 | 0.582 | |
| 0.87 | Sensitivity | 0.725 | 0.686 | 0.627 | 0.608 | 0.588 | 0.647 | 0.667 | 0.647 | 0.608 | 0.667 | 0.627 | 0.667 | 0.647 | |
|  | Specificity | 0.592 | 0.673 | 0.653 | 0.735 | 0.653 | 0.735 | 0.633 | 0.755 | 0.714 | 0.653 | 0.49 | 0.694 | 0.551 | |
|  | Precision | 0.649 | 0.686 | 0.653 | 0.705 | 0.638 | 0.717 | 0.654 | 0.733 | 0.689 | 0.667 | 0.561 | 0.694 | 0.6 | |
| 0.88 | Sensitivity | 0.667 | 0.686 | 0.627 | 0.529 | 0.588 | 0.706 | 0.647 | 0.647 | 0.588 | 0.686 | 0.667 | 0.686 | 0.627 | |
|  | Specificity | 0.612 | 0.694 | 0.673 | 0.653 | 0.633 | 0.714 | 0.633 | 0.735 | 0.694 | 0.694 | 0.531 | 0.714 | 0.551 | |
|  | Precision | 0.642 | 0.7 | 0.667 | 0.614 | 0.625 | 0.72 | 0.647 | 0.717 | 0.667 | 0.7 | 0.596 | 0.714 | 0.593 | |
| 0.89 | Sensitivity | 0.706 | 0.647 | 0.608 | 0.529 | 0.608 | 0.745 | 0.647 | 0.667 | 0.627 | 0.667 | 0.686 | 0.667 | 0.608 | |
|  | Specificity | 0.612 | 0.694 | 0.714 | 0.633 | 0.633 | 0.653 | 0.633 | 0.653 | 0.612 | 0.694 | 0.592 | 0.776 | 0.633 | |
|  | Precision | 0.655 | 0.688 | 0.689 | 0.6 | 0.633 | 0.691 | 0.647 | 0.667 | 0.627 | 0.694 | 0.636 | 0.756 | 0.633 | |
| 0.9 | Sensitivity | 0.745 | 0.627 | 0.627 | 0.529 | 0.569 | 0.765 | 0.667 | 0.686 | 0.667 | 0.686 | 0.686 | 0.686 | 0.647 | |
|  | Specificity | 0.612 | 0.673 | 0.714 | 0.673 | 0.673 | 0.612 | 0.571 | 0.592 | 0.571 | 0.673 | 0.592 | 0.735 | 0.673 | |
|  | Precision | 0.667 | 0.667 | 0.696 | 0.628 | 0.644 | 0.672 | 0.618 | 0.636 | 0.618 | 0.686 | 0.636 | 0.729 | 0.673 | |
| 0.91 | Sensitivity | 0.784 | 0.725 | 0.627 | 0.588 | 0.549 | 0.765 | 0.686 | 0.686 | 0.647 | 0.765 | 0.667 | 0.686 | 0.627 | |
|  | Specificity | 0.592 | 0.592 | 0.694 | 0.673 | 0.694 | 0.653 | 0.571 | 0.531 | 0.612 | 0.633 | 0.612 | 0.714 | 0.673 | |
|  | Precision | 0.667 | 0.649 | 0.681 | 0.652 | 0.651 | 0.696 | 0.625 | 0.603 | 0.635 | 0.684 | 0.642 | 0.714 | 0.667 | |
| 0.92 | Sensitivity | 0.686 | 0.569 | 0.608 | 0.608 | 0.549 | 0.667 | 0.647 | 0.647 | 0.686 | 0.686 | 0.647 | 0.647 | 0.569 | |
|  | Specificity | 0.592 | 0.592 | 0.714 | 0.755 | 0.633 | 0.653 | 0.592 | 0.51 | 0.673 | 0.592 | 0.633 | 0.735 | 0.653 | |
|  | Precision | 0.636 | 0.592 | 0.689 | 0.721 | 0.609 | 0.667 | 0.623 | 0.579 | 0.686 | 0.636 | 0.647 | 0.717 | 0.63 | |
| 0.93 | Sensitivity | 0.706 | 0.686 | 0.647 | 0.667 | 0.569 | 0.667 | 0.588 | 0.647 | 0.706 | 0.706 | 0.647 | 0.627 | 0.647 | |
|  | Specificity | 0.633 | 0.612 | 0.714 | 0.714 | 0.673 | 0.592 | 0.633 | 0.551 | 0.633 | 0.592 | 0.592 | 0.673 | 0.653 | |
|  | Precision | 0.667 | 0.648 | 0.702 | 0.708 | 0.644 | 0.63 | 0.625 | 0.6 | 0.667 | 0.643 | 0.623 | 0.667 | 0.66 | |
| 0.94 | Sensitivity | 0.686 | 0.667 | 0.627 | 0.725 | 0.627 | 0.686 | 0.627 | 0.647 | 0.706 | 0.667 | 0.588 | 0.627 | 0.647 | |
|  | Specificity | 0.592 | 0.612 | 0.714 | 0.694 | 0.714 | 0.531 | 0.592 | 0.51 | 0.592 | 0.592 | 0.551 | 0.653 | 0.653 | |
|  | Precision | 0.636 | 0.642 | 0.696 | 0.712 | 0.696 | 0.603 | 0.615 | 0.579 | 0.643 | 0.63 | 0.577 | 0.653 | 0.66 | |
| 0.95 | Sensitivity | 0.667 | 0.686 | 0.608 | 0.725 | 0.608 | 0.667 | 0.647 | 0.608 | 0.745 | 0.725 | 0.569 | 0.627 | 0.627 | |
|  | Specificity | 0.653 | 0.592 | 0.673 | 0.673 | 0.633 | 0.592 | 0.571 | 0.469 | 0.653 | 0.571 | 0.51 | 0.612 | 0.612 | |
|  | Precision | 0.667 | 0.636 | 0.66 | 0.698 | 0.633 | 0.63 | 0.611 | 0.544 | 0.691 | 0.638 | 0.547 | 0.627 | 0.627 | |
| 0.96 | Sensitivity | 0.647 | 0.686 | 0.569 | 0.686 | 0.627 | 0.549 | 0.627 | 0.627 | 0.608 | 0.529 | 0.549 | 0.608 | 0.569 | |
|  | Specificity | 0.633 | 0.449 | 0.612 | 0.551 | 0.653 | 0.653 | 0.551 | 0.469 | 0.571 | 0.51 | 0.429 | 0.571 | 0.551 | |
|  | Precision | 0.647 | 0.565 | 0.604 | 0.614 | 0.653 | 0.622 | 0.593 | 0.552 | 0.596 | 0.529 | 0.5 | 0.596 | 0.569 | |
| 0.97 | Sensitivity | 0.608 | 0.608 | 0.569 | 0.627 | 0.549 | 0.49 | 0.569 | 0.608 | 0.569 | 0.51 | 0.569 | 0.588 | 0.529 | |
|  | Specificity | 0.551 | 0.184 | 0.531 | 0.531 | 0.735 | 0.592 | 0.592 | 0.633 | 0.449 | 0.327 | 0.449 | 0.633 | 0.51 | |
|  | Precision | 0.585 | 0.437 | 0.558 | 0.582 | 0.683 | 0.556 | 0.592 | 0.633 | 0.518 | 0.441 | 0.518 | 0.625 | 0.529 | |
| 0.98 | In this threshold, no feature got selected in an iteration of LOPOCV. Thus, no model was developed. | | | | | | | | | | | | | |  |

# **Important Features**

**Table F.1: Percentage of times a feature appeared in Leave One Participant Out Cross Validation (LOPOCV) with the variation of the maximum depth of the base estimator.**

| **Feature** | **Depth 3** | **Depth 4** | **Depth 5** | **Depth 6** | **Depth 7** |
| --- | --- | --- | --- | --- | --- |
| Weekday_Communication_Ratio_of_Hamming_6_Hour_Mean | 100 | 99 | 100 | 98 | 92 |
| Weekday_Social_Duration_24_Hour | 7 | 2 | 1 | 1 | 0 |
| Weekday_Education_Duration_24_Hour | 4 | 1 | 0 | 0 | 0 |
| Weekday_Smartphone_Entropy_6_Hour_Mean | 100 | 97 | 75 | 87 | 78 |
| Weekday_Social_Duration_6_Hour_Mean | 2 | 1 | 1 | 0 | 0 |
| Weekday_Smartphone_Ratio_of_Hamming_24_Hour | 5 | 0 | 0 | 0 | 0 |
| Weekend_Smartphone_Ratio_of_Hamming_6_Hour_Mean | 6 | 0 | 7 | 9 | 13 |
| Weekday_Smartphone_Entropy_24_Hour | 100 | 100 | 100 | 100 | 100 |
| Weekday_Smartphone_Entropy_6_Hour_SD | 0 | 0 | 2 | 1 | 1 |
| Weekday_Browser_Duration_6_Hour_SD | 27 | 13 | 12 | 34 | 29 |
| Weekend_Productivity_Duration_24_Hour | 100 | 99 | 98 | 98 | 99 |
| Weekend_Smartphone_Entropy_24_Hour | 100 | 90 | 97 | 95 | 94 |

**Table F.2: The features used for the best set of top-5 classifiers developed based on the filter method Information Gain, wrapper method Boruta, embedded method Random Forest, and Stable method. Here, values present the percentage of times (among all iterations of Leave One Participant Out Cross Validation) a feature was selected by a feature selection approach. Mean denotes the average number of appearances in each FS approach and the features are sorted in descending order based on the mean value. Features are sorted by the mean value. Emb.: Embedded.**

| **Feature** | **Filter** | **Wrapper** | **Emb.** | **Stable** | **Mean** | **Feature** | **Filter** | **Wrapper** | **Emb.** | **Stable** | **Mean** |
| --- | --- | --- | --- | --- | --- | --- | --- | --- | --- | --- | --- |
| Weekday_Smartphone_Entropy_24_Hour | 99 | 100 | 100 | 100 | 99.8 | Weekday_Launcher_#_of_Apps_6_Hour_SD | 6 | 0 | 0 | 0 | 1.5 |
| Weekend_Smartphone_Entropy_24_Hour | 100 | 95 | 100 | 100 | 98.8 | Weekend_launcher_Launch_6_Hour_Mean | 5 | 0 | 0 | 0 | 1.2 |
| Weekday_Smartphone_Entropy_6_Hour_Mean | 100 | 87 | 83 | 100 | 92.5 | Weekday_Tools_Duration_6_Hour_Mean | 0 | 0 | 5 | 0 | 1.2 |
| Weekday_Communication_Ratio_of_Hamming_6_Hour_Mean | 0 | 98 | 96 | 100 | 73.5 | Weekend_Browser_Entropy_24_Hour | 0 | 0 | 5 | 0 | 1.2 |
| Weekday_Browser_Duration_6_Hour_SD | 100 | 34 | 84 | 0 | 54.5 | Weekday_Photo_Video_Entropy_6_Hour_Mean | 0 | 0 | 5 | 0 | 1.2 |
| Weekend_Smartphone_Ratio_of_Hamming_6_Hour_Mean | 0 | 9 | 99 | 97 | 51.2 | Weekday_Tools_Ratio_of_Hamming_6_Hour_SD | 0 | 0 | 5 | 0 | 1.2 |
| Weekend_Productivity_Duration_24_Hour | 0 | 98 | 100 | 7 | 51.2 | Weekend_Communication_Duration_6_Hour_SD | 0 | 0 | 3 | 1 | 1 |
| Weekday_Education_Duration_24_Hour | 0 | 0 | 66 | 100 | 41.5 | Weekend_Smartphone_Duration_24_Hour | 0 | 0 | 3 | 1 | 1 |
| Weekday_Tools_Entropy_24_Hour | 0 | 0 | 18 | 99 | 29.2 | Weekend_Browser_Duration_6_Hour_Mean | 0 | 0 | 2 | 2 | 1 |
| Weekend_Photo_Video_#_of_Apps_6_Hour_SD | 0 | 0 | 7 | 100 | 26.8 | Weekday_Social_Duration_6_Hour_SD | 0 | 0 | 4 | 0 | 1 |
| Weekend_Communication_Entropy_6_Hour_SD | 100 | 0 | 4 | 0 | 26 | Weekday_Smartphone_Total_#_of_Sessions_6_Hour_SD | 4 | 0 | 0 | 0 | 1 |
| Weekend_Photo_Video_#_of_Apps_24_Hour | 1 | 0 | 0 | 100 | 25.2 | Weekday_Productivity_Ratio_of_Hamming_24_Hour | 3 | 0 | 0 | 0 | 0.8 |
| Weekend_Smartphone_Review_Session_#_6_Hour_SD | 100 | 0 | 0 | 0 | 25 | Weekday_Tools_Launch_6_Hour_SD | 0 | 0 | 3 | 0 | 0.8 |
| Weekend_Social_Launch_6_Hour_SD | 0 | 0 | 0 | 98 | 24.5 | Weekend_Browser_Duration_24_Hour | 0 | 0 | 1 | 2 | 0.8 |
| Weekday_Smartphone_Entropy_6_Hour_SD | 67 | 1 | 15 | 0 | 20.8 | Weekend_Social_Launch_24_Hour | 0 | 0 | 0 | 3 | 0.8 |
| Weekday_Social_Duration_6_Hour_Mean | 0 | 0 | 83 | 0 | 20.8 | Weekday_Browser_Entropy_24_Hour | 0 | 0 | 3 | 0 | 0.8 |
| Weekday_Photo_Video_Ratio_of_Hamming_24_Hour | 0 | 0 | 1 | 81 | 20.5 | Weekend_Social_Launch_6_Hour_Mean | 0 | 0 | 0 | 3 | 0.8 |
| Weekend_Productivity_Entropy_24_Hour | 60 | 0 | 0 | 0 | 15 | Weekday_Smartphone_Duration_24_Hour | 0 | 0 | 3 | 0 | 0.8 |
| Weekday_Social_Duration_24_Hour | 0 | 1 | 55 | 0 | 14 | Weekend_Tools_Duration_6_Hour_Mean | 0 | 0 | 3 | 0 | 0.8 |
| Weekday_Smartphone_Ratio_of_Hamming_24_Hour | 0 | 0 | 44 | 0 | 11 | Weekday_Communication_#_of_Apps_24_Hour | 3 | 0 | 0 | 0 | 0.8 |
| Weekend_Browser_#_of_Apps_6_Hour_SD | 36 | 0 | 1 | 0 | 9.2 | Weekday_Social_Launch_6_Hour_Mean | 0 | 0 | 2 | 0 | 0.5 |
| Weekend_Productivity_Launch_24_Hour | 29 | 0 | 0 | 0 | 7.2 | Weekend_Smartphone_Review_Session_#_24_Hour | 2 | 0 | 0 | 0 | 0.5 |
| Weekend_Tools_Duration_24_Hour | 0 | 0 | 22 | 0 | 5.5 | Weekday_Browser_Duration_6_Hour_Mean | 0 | 0 | 2 | 0 | 0.5 |
| Weekday_Browser_Duration_24_Hour | 0 | 0 | 21 | 0 | 5.2 | Weekend_Smartphone_Micro_Use_#_6_Hour_Mean | 0 | 0 | 2 | 0 | 0.5 |
| Weekday_Communication_Launch_6_Hour_Mean | 0 | 0 | 19 | 0 | 4.8 | Weekend_Photo_Video_Launch_24_Hour | 2 | 0 | 0 | 0 | 0.5 |
| Weekday_Communication_Duration_6_Hour_Mean | 0 | 0 | 18 | 0 | 4.5 | Weekend_Browser_#_of_Apps_6_Hour_Mean | 0 | 0 | 2 | 0 | 0.5 |
| Weekday_Photo_Video_Launch_6_Hour_SD | 0 | 0 | 16 | 1 | 4.2 | Weekday_Education_Launch_24_Hour | 1 | 0 | 1 | 0 | 0.5 |
| Weekend_Tools_Duration_6_Hour_SD | 0 | 0 | 16 | 0 | 4 | Weekday_Photo_Video_#_of_Apps_24_Hour | 2 | 0 | 0 | 0 | 0.5 |
| Weekday_Social_Launch_6_Hour_SD | 0 | 0 | 16 | 0 | 4 | Weekend_Smartphone_Ratio_of_Hamming_6_Hour_SD | 0 | 0 | 1 | 0 | 0.2 |
| Weekend_Tools_Ratio_of_Hamming_6_Hour_SD | 15 | 0 | 0 | 0 | 3.8 | Weekday_Productivity_Duration_24_Hour | 0 | 0 | 1 | 0 | 0.2 |
| Weekend_Productivity_#_of_Apps_24_Hour | 13 | 0 | 0 | 0 | 3.2 | Weekend_Tools_Launch_6_Hour_SD | 0 | 0 | 1 | 0 | 0.2 |
| Weekend_Communication_Launch_24_Hour | 0 | 0 | 12 | 0 | 3 | Weekday_Tools_Launch_6_Hour_Mean | 0 | 0 | 1 | 0 | 0.2 |
| Weekday_Smartphone_Review_Session_#_6_Hour_Mean | 12 | 0 | 0 | 0 | 3 | Weekend_Smartphone_Duration_6_Hour_Mean | 0 | 0 | 0 | 1 | 0.2 |
| Weekday_Communication_#_of_Apps_6_Hour_SD | 10 | 0 | 1 | 0 | 2.8 | Weekday_Browser_Launch_6_Hour_Mean | 0 | 0 | 1 | 0 | 0.2 |
| Weekday_Social_Launch_24_Hour | 0 | 0 | 9 | 0 | 2.2 | Weekday_Smartphone_Review_Session_#_24_Hour | 1 | 0 | 0 | 0 | 0.2 |
| Weekday_Games_#_of_Apps_24_Hour | 9 | 0 | 0 | 0 | 2.2 | Weekday_Browser_Launch_24_Hour | 0 | 0 | 1 | 0 | 0.2 |
| Weekday_Tools_Ratio_of_Hamming_6_Hour_Mean | 0 | 0 | 0 | 8 | 2 | Weekday_Launcher_Duration_6_Hour_Mean | 0 | 0 | 1 | 0 | 0.2 |
| Weekday_Productivity_Launch_24_Hour | 0 | 0 | 8 | 0 | 2 | Weekend_Smartphone_Micro_Use_#_24_Hour | 0 | 0 | 1 | 0 | 0.2 |
| Weekend_launcher_Launch_24_Hour | 8 | 0 | 0 | 0 | 2 | Weekend_Tools_Launch_24_Hour | 0 | 0 | 1 | 0 | 0.2 |
| Weekday_Smartphone_Ratio_of_Hamming_6_Hour_SD | 0 | 0 | 7 | 0 | 1.8 | Weekday_Smartphone_Launch_6_Hour_SD | 0 | 0 | 1 | 0 | 0.2 |
| Weekday_Communication_Duration_24_Hour | 0 | 0 | 7 | 0 | 1.8 | Weekday_Smartphone_Micro_Use_#_6_Hour_SD | 0 | 0 | 1 | 0 | 0.2 |
| Weekday_Communication_Ratio_of_Hamming_6_Hour_SD | 6 | 0 | 0 | 0 | 1.5 | Weekend_Communication_#_of_Apps_6_Hour_Mean | 1 | 0 | 0 | 0 | 0.2 |
| Weekend_Browser_Duration_6_Hour_SD | 0 | 0 | 6 | 0 | 1.5 | Weekend_Photo_Video_Ratio_of_Hamming_24_Hour | 0 | 0 | 0 | 1 | 0.2 |
| Weekday_Games_Duration_24_Hour | 5 | 0 | 0 | 1 | 1.5 | Weekend_Tools_Entropy_24_Hour | 0 | 0 | 1 | 0 | 0.2 |

**Table F.3: Features selected when using the threshold 0.97 in Stable FS. LOPOCV: Leave One Participant Out Cross Validation.**

| **Feature** | **Percentage of times a feature appeared in LOPOCV** |
| --- | --- |
| Weekend_Photo_Video_#_of_Apps_24_Hour | 5 |
| Weekday_Communication_Ratio_of_Hamming_6_Hour_Mean | 98 |
| Weekday_Smartphone_Entropy_24_Hour | 28 |
